# Haemosiderotic Synovitis Secondary to Anticoagulant Use: An Unusual Mechanism of Failure of a Unicompartmental Knee Replacement

**DOI:** 10.1155/2019/3959278

**Published:** 2019-11-14

**Authors:** Jonathan Bartlett, Surjit S. Lidder, Andrew T. Bucknill

**Affiliations:** ^1^Corpus Christi College, Cambridge CB2 1RH, UK; ^2^Department of Orthopaedic Surgery, The Royal Melbourne Hospital, Melbourne, VIC 3050, Australia

## Abstract

Haemosiderotic synovitis is a rare condition caused by recurrent or chronic haemarthroses. This may lead to intra-articular destruction, a painful joint, and, if untreated, ankylosis of the joint. We highlight a case of an elderly lady who presented to an orthopaedic clinic with left knee pain, following recurrent left knee atraumatic haemarthroses secondary to oral anticoagulant use. At her presentation, she had a left medial unicompartmental knee prosthesis in situ. Weight bearing radiographs of the left knee showed marked loss of lateral joint space with valgus alignment. These radiographic findings were not present on the radiographs taken at her first presentation with haemarthrosis nine months previously. A left revision total knee arthroplasty was performed, and a diagnosis of haemosiderotic synovitis was made following histological analysis of intraoperative tissue samples. This case highlights an unusual mechanism of failure of a unicompartmental knee replacement. Though haemosiderotic synovitis is an exceedingly rare condition, it must be considered following recurrent haemarthrosis as, due to its destructive nature, prompt recognition and treatment is paramount.

## 1. Introduction

Haemosiderotic synovitis is a proliferative synovial disorder that develops as the result of recurrent or chronic haemarthroses [1–4]. Although exceedingly rare in nonhaemophiliac populations, oral anticoagulant use amongst other causes has been documented in the literature [3]. Haemosiderotic synovitis most commonly affects the knee joint, owing to this joint's propensity to articular haemorrhage [1]. The synovial proliferation induced through haemarthrosis, theorised to be the results of the iron-induced mdm2 and c-myc oncogene expression and increases in VEGF levels, leads to a hypertrophic and hyperaemic synovium more susceptible to haemorrhage, thus establishing a cycle of recurrent inflammation and haemarthroses [5–7]. Chronic haemosiderotic synovitis can result in intra-articular synovial fibrosis, capsular fibrosis, and cartilage destruction, ultimately leading to an ankylosed, dysfunctional joint [4, 8]. We describe a case of nonhaemophiliac haemosiderotic synovitis following recurrent warfarin-related atraumatic haemarthrosis in a woman presenting with worsening left knee pain and a previous unicompartmental left knee prosthesis.

## 2. Case Presentation

A 77-year-old woman presented to an orthopaedic clinic with an eight-month history of pain in her left knee and recurrent haemarthroses within this joint whilst taking warfarin. She had uncomplicated bilateral unicompartmental knee replacements with the left in 2010 and the right in 2008. Over the previous nine months, she was admitted to the Emergency Department four times with haemarthrosis of the left knee joint and supratherapeutic International Normalised Ratios (INR), each time improving with conservative management. The most recent haemarthrosis, one month previously, was subsequently aspirated. No bacterial cultures were grown from this sample, and a Synovasure Alpha Defensin Test was negative. At the time of her presentation to clinic, she had been on warfarin for five years following recurrent deep vein thromboses and a pulmonary embolus. Her past medical history was notable for insulin managed type 2 diabetes mellitus, hypertension, hyperlipidaemia, and chronic renal impairment. Although initially able to mobilise with a four-wheeled frame, when she presented to the clinic, her mobility had deteriorated and was unable to weight bear, using a wheelchair to mobilise. Examination showed a well-healed surgical scar, an antalgic gait, mild knee swelling, and a partially correctable 20° valgus deformity of the left knee. Her range of movement was 0-90°.

Her INR was 1.9 (therapeutic range 2.0–3.0). Haemoglobin was 13.6 (normal range: 11.7–15.5 g/dL). White Cell Count was 7.4 (normal range: 4.1–11.2 × 10^3^/*μ*L), and CRP was 3.5 (normal range: 1–5 mg/L). Urea and electrolytes were within normal range.

**Figure 1:**
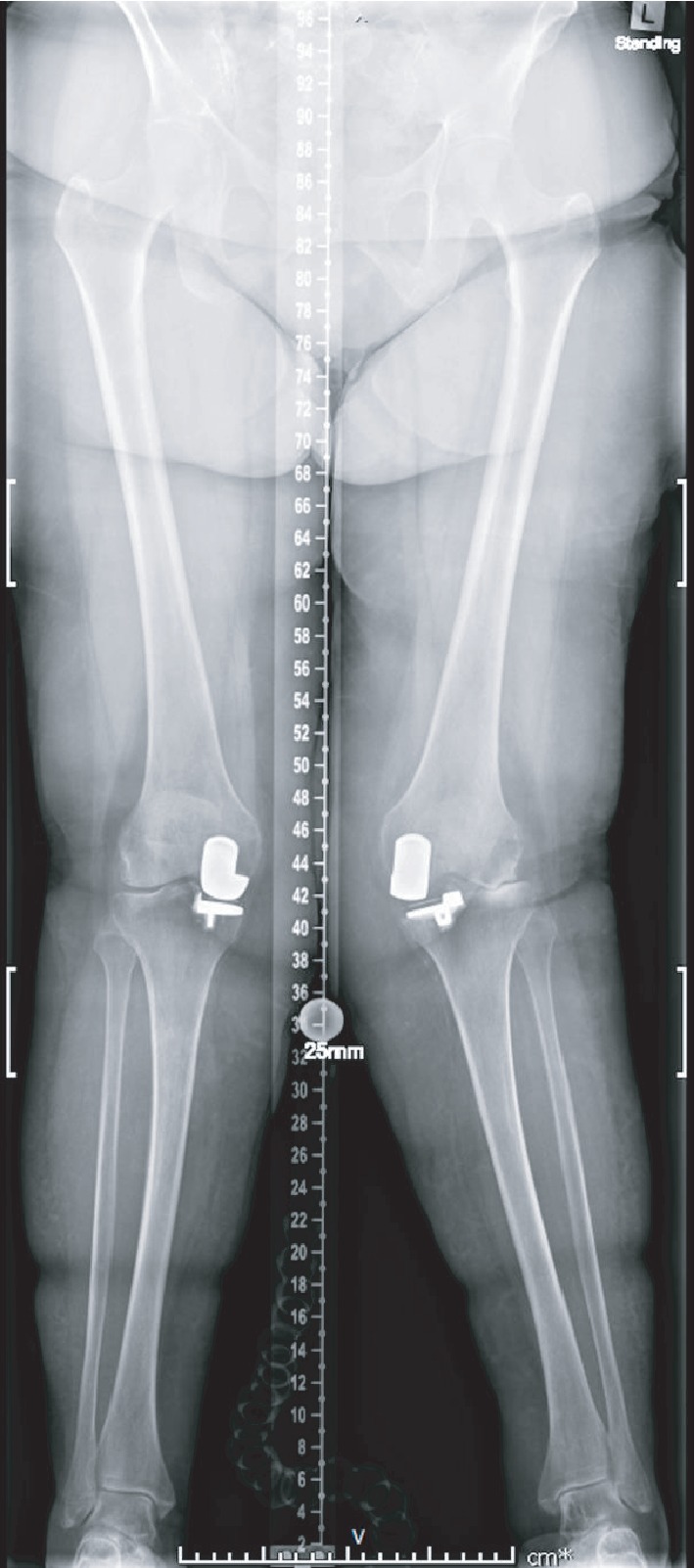
Long-leg radiographs demonstrating bilateral medial unicompartmental knee replacements. Valgus deformity of the left knee with loss of lateral joint space.

**Figure 2:**
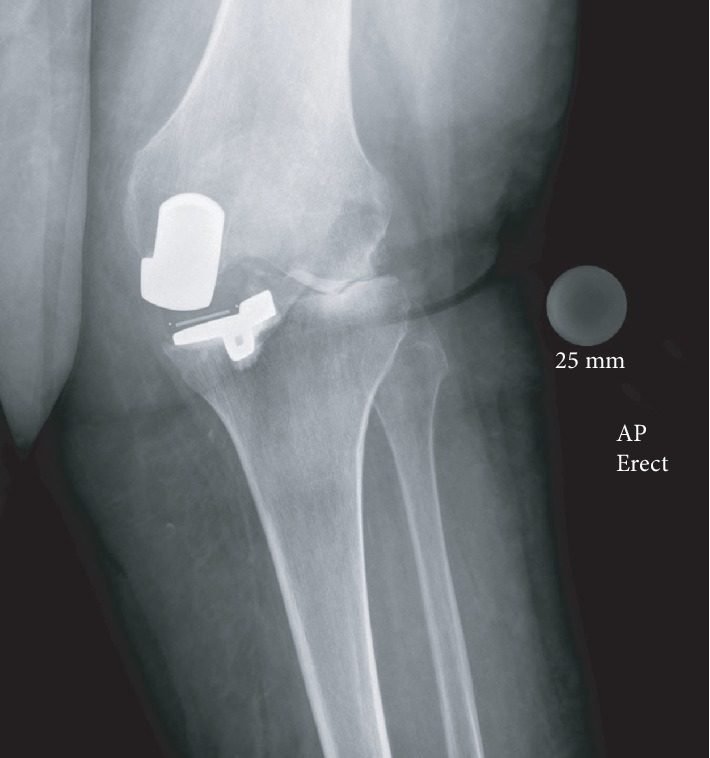
Weight-bearing AP radiograph of the left knee. Medial unicompartmental knee replacement in situ. Loss of lateral joint space, subchondral sclerosis, and infarction of the lateral femoral condyle.

**Figure 3:**
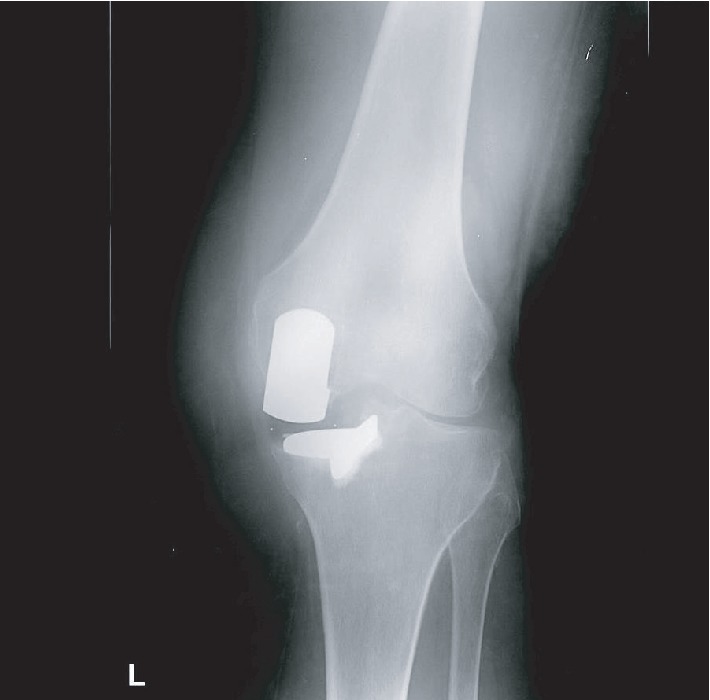
Weight-bearing AP radiograph of the left knee from nine months prior to presentation. Medial unicompartmental knee replacement in situ.

Weight-bearing long-leg radiographs demonstrated bilateral unicompartmental knee replacements. There was a prominent valgus (20°) deformity of the left knee with loss of lateral compartment joint space with associated subchondral sclerosis and infarction of the lateral femoral condyle (Figures 1 and 2). These radiographic findings were not present on a radiograph taken at her first presentation with haemarthrosis nine months previously (Figure 3).

**Figure 4:**
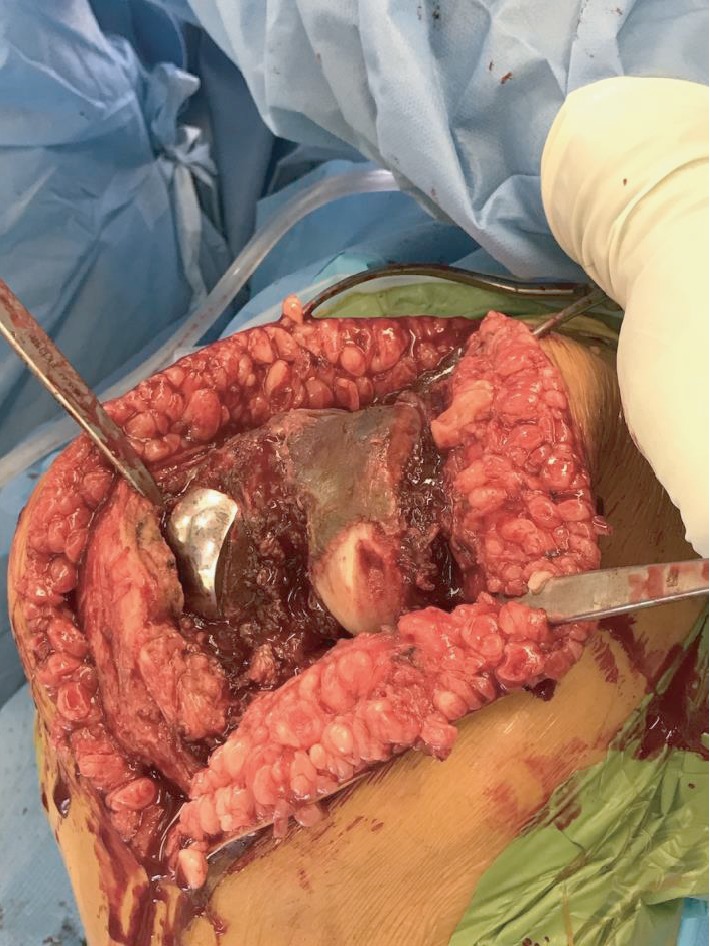
Clinical photo of the left knee with a lateral parapatellar exposure. Medial unicompartmental knee replacement in situ. Haemosiderin staining of the lateral femoral condyle.

Following discussion with the haematologist, warfarin was stopped and bridging Clexane used. The patient underwent revision of the left unicompartmental knee to a total knee replacement through a lateral parapatella approach. Intraoperatively, computer navigation was utilised (Brainlab, Munich, Germany) to aid alignment and ligament balancing. A short-stemmed tibial component was used and a posterior stabilised knee component. The lateral femoral condyle was delaminated, and the cartilage was haemosiderin stained (Figure 4). Intraoperative samples confirmed no infection, and histology confirmed the diagnosis of haemosiderotic synovitis.

**Figure 5:**
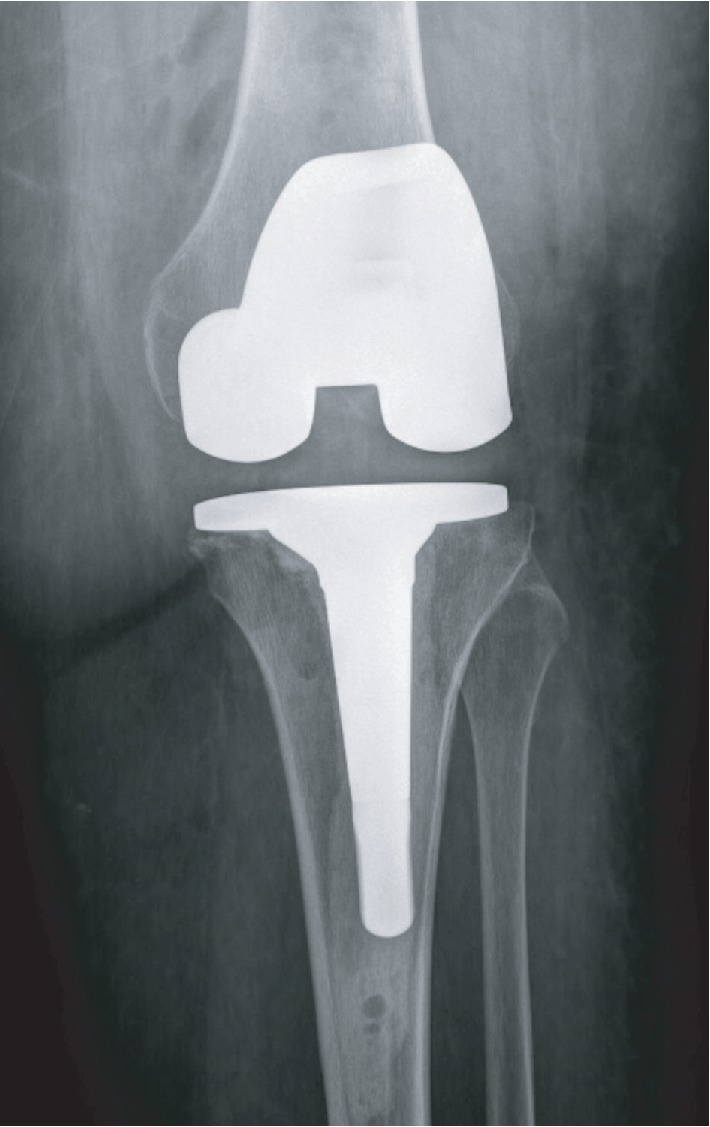
Postoperative AP radiograph of the left knee. Revision left total knee in situ. Pin holes used for navigation are present distal to the stemmed tibial component.

**Figure 6:**
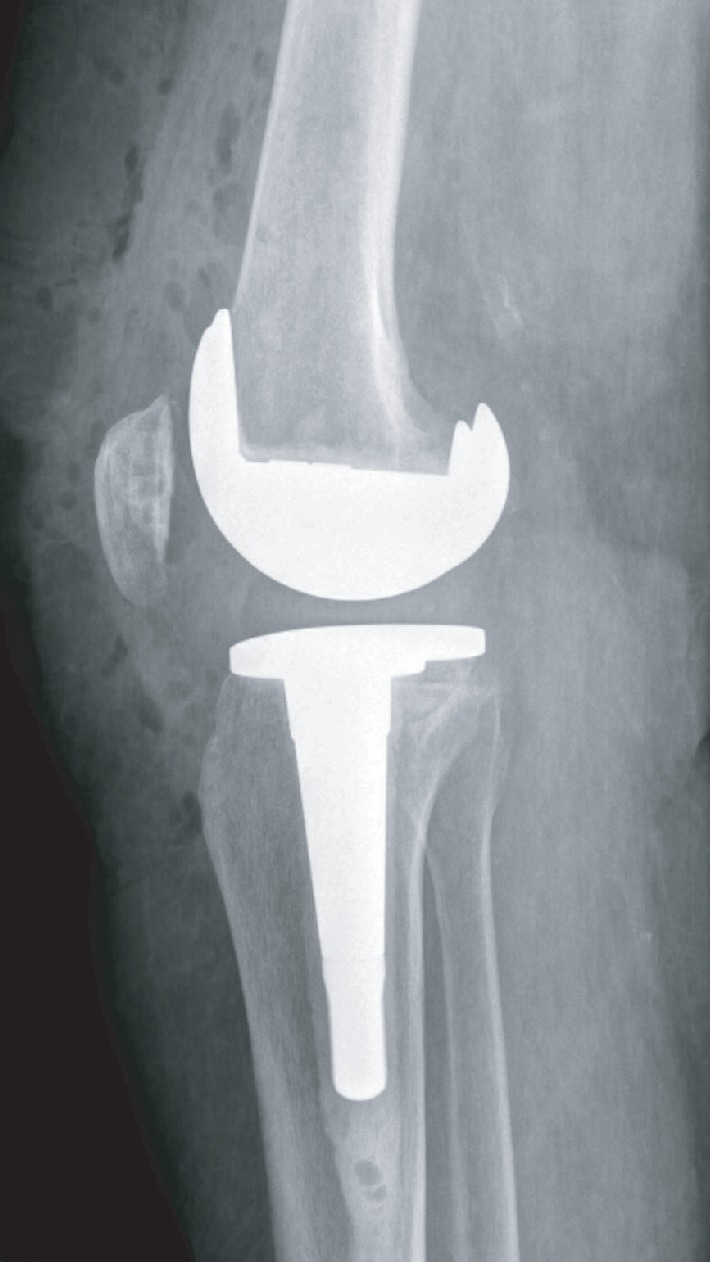
Postoperative lateral radiograph of the left knee. Revision left total knee in situ. Pin holes used for navigation are present distal to the stemmed tibial component.

**Figure 7:**
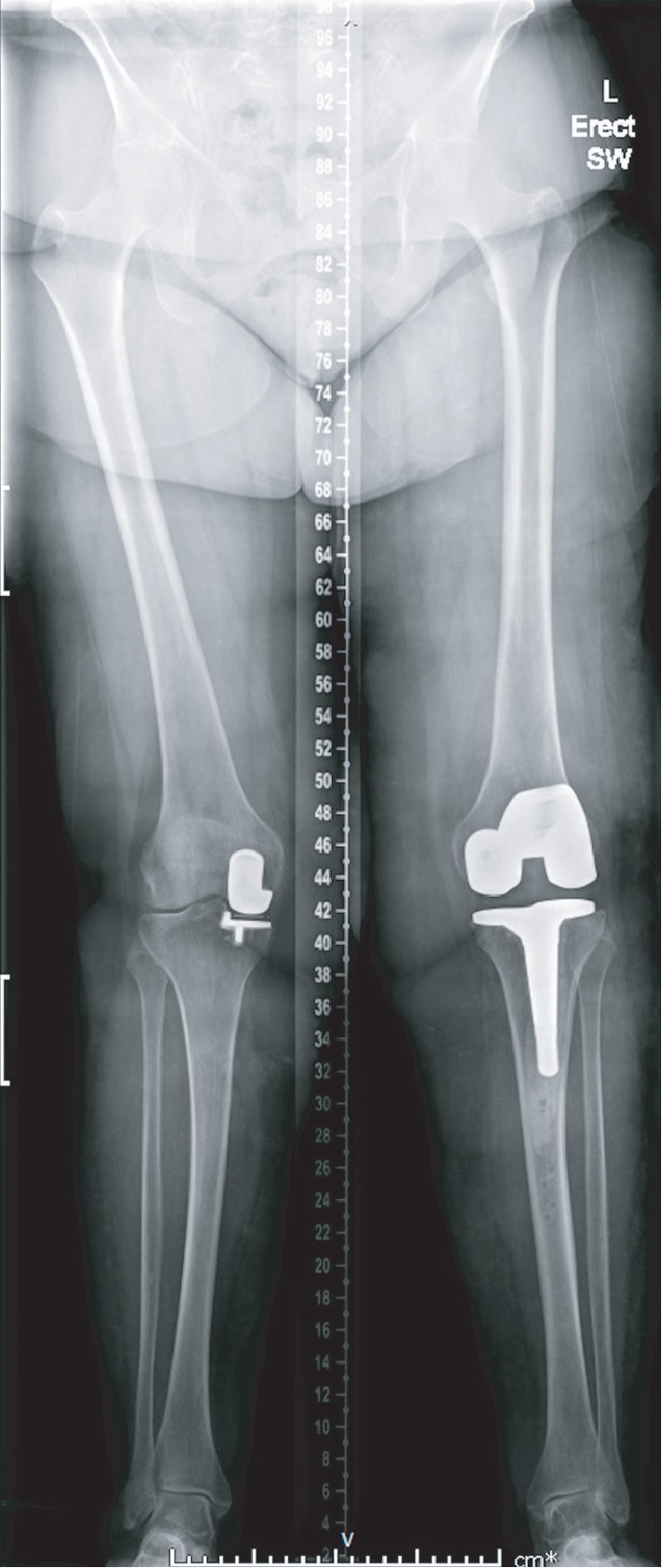
Long-leg radiographs demonstrating a medial unicompartmental knee replacement and revision left total knee replacement at six months.

Postoperatively, the patient had made good progress at six weeks. She had no pain and a stable knee through a flexion of 0-100°. At six months of follow-up, she was walking with a single point stick at home and managed to go shopping with her husband. Her knee was stable with flexion maintained at 0-100°. Oxford knee scores had improved from 17 preoperatively to 38 at six months. Radiographs showed good alignment of the revision left total knee replacement (Figures 5–7). Informed written consent was obtained from the patient for production of this case report.

## 3. Discussion

In this case, we highlight an unusual mechanism of failure of a unicompartmental knee replacement. Here, recurrent haemarthroses in a patient on oral anticoagulant were complicated by haemosiderotic synovitis. The most common causes of failure of unicompartmental failure knee replacements vary depending on the bearing design. With fixed bearings, polyethylene wear and aseptic loosening constitute the majority of cases necessitating conversion to total knee arthroplasty, whereas with mobile bearings, bearing dislocation is the most common indication for revision [9]. Another common mode of failure of a unicompartmental knee replacement—progression of osteoarthritis—is excluded by the radiographs taken at her first presentation with haemarthroses nine months previously (Figure 3).

Patients with haemosiderotic synovitis commonly present with pain and stiffness of the involved joint, often due to secondary osteoarthritis development [1, 10]. However, these symptoms may also represent severe osteoarthritis, rheumatoid arthritis, or pigmented villonodular synovitis, thus making a clinical diagnosis of haemosiderotic synovitis extremely difficult [3, 10]. As found with this patient, a history of recurrent haemarthrosis is a key component of the disease pathology and is most commonly the result of inherited clotting factor deficiencies [1, 10].

In haemosiderotic synovitis, recurrent, or chronic, haemarthroses induce synovial proliferation and establish a vicious cycle of recurrent inflammation and haemarthrosis [5–7]. Though the pathology of haemosiderotic synovitis has not been fully characterised, it is theorised that iron-induced angiogenesis through VEGF signalling and mdm2 and c-myc oncogene upregulation results in the development of a hyperaemic and hypertrophic synovium more susceptible to haemorrhage. Although this disease entity is exceedingly rare in nonhaemophiliac populations, other documented causes include oral anticoagulant use, trauma, rheumatoid or psoriatic arthritis, collagen vascular disease, and myeloproliferative disorders [1]. Chronic haemosiderotic synovitis ultimately results in intra-articular synovial fibrosis, capsular fibrosis, and cartilage destruction leading to an ankylosed, dysfunctional joint [4, 8, 11].

The diagnosis of haemosiderotic synovitis can only be made histologically following sampling of the synovium [10]. Accumulation of haemosiderin leads to rusty brown discolouration of the tissue and loss of the normal glistening translucent appearance of the synovial membrane [12]. Though this gross macroscopic appearance is often indistinguishable from pigmented villonodular synovitis, in haemosiderotic synovitis, there is no proliferation of mononuclear synovial cells, lipid-laden cells, or multinucleated osteoclast-like giant cells [1, 10, 12]. As such, microscopic examination is essential to confirm the diagnosis.

Though rare, cases of nonhaemophiliac haemosiderotic synovitis have been noted in the literature involving several joints [1, 3, 10]. Yalçin et al. described a case of a 20-year-old female presenting with recurrent atraumatic joint effusions which, following histological analysis, was found to be the result of haemosiderotic synovitis and was successfully treated arthroscopically [10]. Furthermore, a previous case report described a case of haemosiderotic synovitis presenting as pain and swelling of the left knee [1]. Interestingly, in this case, there was no history of bleeding diatheses or anticoagulant use and the cause of this patient's haemosiderotic synovitis was unclear. Though the knee is the most common site of haemosiderotic synovitis, due to its susceptibility to haemarthroses, this condition has been documented to affect other joints, including the shoulder [3].

Once a diagnosis of haemosiderotic synovitis is suspected, prompt treatment is paramount. Due to paucity of documented cases of haemosiderotic synovitis, the most effective treatment option remains unclear, and no studies have assessed the effectiveness of therapies in nonhaemophiliac populations. Radiosynovectomy, arthroscopic synovectomy, and radio-isotopic joint injection, aimed at reducing the frequency and intensity of haemarthroses, have been suggested to be effective in the initial management of haemosiderotic synovitis in patients with haemophilia [11, 13–15]. Total knee arthroplasty is indicated if there is loss of joint space, severe pain, or evidence of deformity.

Importantly, as many of these patients have complex haematological needs, a multidisciplinary approach is paramount. Management of coagulation deficits or anticoagulation is essential to prevent further haemarthrosis or the development of chronic haemarthrosis. Additionally, it is important to consider the preoperative and postoperative haematological needs of these patients and monitor their clotting profiles closely to prevent complications such as bleeding or thromboembolic events.

Although rare, haemosiderotic synovitis is an important consideration in patients presenting with knee pain following haemarthroses. Suspicion should further be raised if there is a history of haemophilia or supratherapeutic oral anticoagulant use. Given the destructive nature of this condition and the risk of ankylosing deformity, prompt recognition and treatment with a multidisciplinary approach is paramount.

## References

[1] Jayalakshmi V., Chikhale N. P., Mishra A., Cherian S. (2014). Nonhemophilic hemosiderotic synovitis of the knee: a case report and review of literature. *Indian Journal of Pathology and Microbiology*.

[2] Mahendra G., Kliskey K., Athanasou N. A. (2010). Immunophenotypic distinction between pigmented villonodular synovitis and haemosiderotic synovitis. *Journal of Clinical Pathology*.

[3] France M. P., Gupta S. K. (1991). Nonhemophilic hemosiderotic synovitis of the Shoulder. *Clinical Orthopaedics and Related Research*.

[4] Nacca C. R., Harris A. P., Tuttle J. R. (2017). Hemophilic arthropathy. *Orthopedics*.

[5] Acharya S. S., Kaplan R. N., Macdonald D., Fabiyi O. T., DiMichele D., Lyden D. (2011). Neoangiogenesis contributes to the development of hemophilic synovitis. *Blood*.

[6] Wen F.-Q., Jabbar A. A., Chen Y.-X., Kazarian T., Patel D. A., Valentino L. A. (2002). c-myc proto-oncogene expression in hemophilic synovitis: in vitro studies of the effects of iron and ceramide. *Blood*.

[7] Bhat V., Olmer M., Joshi S. (2015). Vascular remodeling underlies re-bleeding in hemophilic arthropathy. *American Journal of Hematology*.

[8] Melchiorre D., Manetti M., Matucci-Cerinic M. (2017). Pathophysiology of hemophilic arthropathy. *Journal of Clinical Medicine*.

[9] Vasso M., Corona K., D’Apolito R., Mazzitelli G., Panni A. (2017). Unicompartmental knee arthroplasty: modes of failure and conversion to total knee arthroplasty. *Joints*.

[10] Yalçin N., Bektaşer B., Ciçekli O., Uğraş S., Doğan M. (2010). An unusual cause of recurrent joint effusions: nonhemophilic hemosiderotic synovitis of the knee. *Acta Orthopaedica et Traumatologica Turcica*.

[11] Rodriguez-Merchan E. C. (2014). The knee in severe haemophilia with special emphasis on surgical/invasive procedures. *Thrombosis Research*.

[12] Roosendaal G., Mauser-Bunschoten E. P., De Kleijn P. (1998). Synovium in haemophilic arthropathy. *Haemophilia*.

[13] Rodriguez-Merchan E. C. (2014). Hemophilic synovitis of the knee: radiosynovectomy or arthroscopic synovectomy?. *Expert Review of Hematology*.

[14] Rodriguez-Merchan E. C., De La Corte-Rodriguez H., Jimenez-Yuste V. (2014). Is radiosynovectomy (RS) effective for joints damaged by haemophilia with articular degeneration in simple radiography (ADSR)?. *Thrombosis Research*.

[15] Ohdera T., Tokunaga M., Hiroshima S., Yoshimoto E., Matsuda S. (2004). Recurrent hemarthrosis after knee joint arthroplasty: etiology and treatment. *The Journal of Arthroplasty*.

